# Heart failure with preserved ejection fraction—Out with the old and out with the new?

**DOI:** 10.3389/fcvm.2022.943572

**Published:** 2022-07-27

**Authors:** Luis Tolento Cortes, Lisa Hong

**Affiliations:** Loma Linda University School of Pharmacy, Loma Linda, CA, United States

**Keywords:** heart failure, preserved ejection fraction, HFpEF, pharmacology, heart failure management

## Introduction

Reported 5-year mortality rates among patients diagnosed with heart failure are upwards of 50% (1, 2). Recent studies have demonstrated similar mortality rates between patients with reduced ejection fraction (HFrEF; EF ≤ 40%), mildly reduced ejection fraction (HFmrEF; EF 41%-49%) and preserved ejection fraction (HFpEF; EF ≥50%) (2, 3). While most guideline recommendations regarding pharmacotherapy pertain to patients with HFrEF, the above observation suggests a need for mortality-reducing therapy in all subtypes of heart failure (4, 5).

In the 2021 ESC update, advances in the pharmacological management of HFpEF are discussed and conclude that there are currently no convincing studies supporting morbidity/mortality benefits with HFpEF treatment as all studies have failed to achieve their primary endpoints (4). As previously accepted, the management of patients with HFpEF revolves around acute symptom management with agents such as diuretics in addition to the management of chronic comorbidities that may contribute to the progression of heart failure. However, medication management of HFpEF has recently been reassessed with some newer studies assessing the utility of various therapies in this population. Notably, these trials were conducted in patients with LVEF as low as 40% complicating the generalizability of results to patients with HFpEF as many of the study subjects would otherwise be categorized under HFmrEF. A brief synopsis of the results from select trials are summarized below and in Table 1.

**Table 1:** Summary of select clinical trials.

**Trial**	**Interventions**	**Population**	**Primary outcomes**	**Results**
PEP-CHF (6)	Perindopril vs. placebo	Adults ≥70 years old with HF, on diuretics with an ECG suggestive of diastolic dysfunction but LV wall motion index of 1.4 and LVEF ≥40%	Mortality or unplanned HF-related hospitalization in 1 year	23.6 vs. 25.1% HR 0.92 (95% CI 0.70–1.21; *p* = 0.545)
CHARM-preserved (7)	Candesartan vs. placebo	Adults ≥18 years old, NYHA class II–IV, history of HF hospitalization and LVEF >40%	Cardiovascular death or HF-related admission	22 vs. 24.3% Adjusted HR 0.86 (95% CI 0.74–1.00; *p* = 0.051)
I-PRESERVE (8)	Irbesartan vs. placebo	Adults ≥60 years old with HF symptoms, LVEF ≥45% and HF hospitalization in 6 months or evidence of HF or substrate of diastolic heart failure	Mortality and cardiovascular related hospitalizations	35.8% vs. 37% HR 0.95 (95% CI 0.86–1.05; *p* = 0.35)
PARAMOUNT-HF (9)	Sacubitril/valsartan vs. valsartan	Adults ≥40 years old, LVEF 45%, heart failure signs/symptoms, NT-proBNP >400 pg/ml, on diuretic therapy, SBP >140 mmHg or 160 mmHg if on ≥3 BP medications, eGFR ≥30 ml/min/1.73 m^2^ and K <5.2 mmol/L	Change in NT-proBNP at 12 weeks	Change from baseline 22.7 vs. 3.2% Ratio of change 0.77 (95% CI 0.64–0.92; *p* = 0.005)
PARAGON-HF (10)	Sacubitril/valsartan vs. valsartan	Adults ≥50 years old, signs/symptoms of HF, NYHA class II–IV, EF ≥45% in last 6 months, elevated natriuretic peptides, structural heart disease and on diuretics.	Hospitalizations for HF and death from cardiovascular causes	37.1 vs. 42.2% RR 0.87 (95% CI 0.75–1.01; *p* = 0.06)
SENIORS (11)	Nebivolol vs. placebo	Adults ≥ 70 years old, LVEF≥40%	All-cause mortality or cardiovascular related hospitalization	31.1 vs. 25.3% HR 0.86 (95% CI 0.75–0.99; *p* = 0.04)
Aldo-DHF (12)	Spironolactone vs. placebo	Adults ≥ 50 years old, LVEF ≥50%, NYHA II–III, peak VO_2_ ≤ 25 ml/min/kg, diastolic dysfunction on ECG or atrial fibrillation	Changes in diastolic function (Mean estimate of filling pressure improvement) and maximal exercise capacity (Mean Peak VO_2_)	Diastolic function 12.1 vs. 13.6 Difference −1.5 (95% CI −2.0 to −0.9; *p* < 0.001) Exercise capacity 16.8 vs. 16.9 Difference 0.01 (95% CI −0.6 to 0.8; *p* = 0.81)
TOPCAT (13)	Spironolactone vs. placebo	Adults ≥50 years old, LVEF 45%, 1 HF sign/symptom, HF hospitalization within 1 year or BNP ≥100 pg/ml or NT-proBNP ≥360 pg/ml	Cardiovascular death, cardiac arrest or HF-related hospitalization	18.6 vs. 20.4% HR 0.89 (95% CI 0.77–1.04; *p* = 0.14)
EMPEROR PRESERVED (14)	Empagliflozin vs. placebo	Adults ≥18 years old, NYHA class II-IV, LVEF >40%, HF hospitalization in last 12 months or structural heart disease within 6 months, NT-proBNP ≥300 pg/ml without atrial fibrillation and on stable dose of diuretics	Cardiovascular death or HF-related hospitalization	13.8% vs. 17.1% HR 0.79 (95% CI 0.69–0.90; *p* < 0.001)

## Role of ACEI/ARB/ARNI therapy

The role of ACEI/ARB therapy in HFpEF stems from the PEP-CHF (perindopril), I-PRESERVE (irbesartan) and CHARM-Preserved (candesartan) studies. The PEP-CHF trial did not show any statistically significant differences in mortality/HF-related hospitalizations between those who received perindopril vs. placebo and was underpowered for the primary outcome. Notably, the study showed a benefit in the reduction of HF hospitalizations favoring perindopril at 1 year, but this benefit was not sustained as the difference was negligible when compared to placebo thereafter (6). The CHARM-Preserved trial reported that patients who received candesartan vs. placebo had a reduction in HF-related admissions after covariate adjustment (*p* = 0.072 before adjustment vs. *p* = 0.047 after adjustment) (7). Though, for the composite primary outcome including CV-related death and HF-related admissions, the observed difference was not significant despite covariate adjustment. Furthermore, the I-PRESERVE trial failed to find a difference in mortality or cardiovascular admissions in patients who received irbesartan vs. placebo (8). Reduction in HF hospitalization was seen in only one of these three trials, which had the most patients with HFmrEF and improved outcomes associated with use of ACEI/ARB therapy in HFpEF are likely derived from their benefit in the management of common comorbidities such as hypertension.

The role of ARNI in HFpEF was assessed in the PARAMOUNT-HF and PARAGON-HF trials. These trials compared sacubitril/valsartan vs. valsartan. While the PARAMOUNT-HF trial demonstrated a reduction in NT-proBNP (a marker for LV wall stress), the clinical relevance of this surrogate outcome is not clear and the PARAGON-HF trial demonstrated no difference in cardiovascular deaths or HF hospitalizations (9, 10). A subgroup analysis in the PARAGON-HF trial suggested a reduction in hospitalizations in patients with a LVEF ≤ 57% and sacubitril/valsartan carries an FDA-approved indication for HFpEF based on these results. The subgroup analysis included patients who would be categorized under HFmrEF but only a limited number of those who would fall within the parameters for HFpEF. The inclusion of patients with HFmrEF in these results precludes the ability to conclude the same benefit with sacubitril/valsartan exclusively among patients with HFpEF. Despite these data, there is insufficient evidence to support a strong recommendation for ARNI therapy in patients with HFpEF at this time. However, for patients with other chronic diseases where an ARB is indicated, ARNI therapy may be reasonable to consider, provided the patient can afford it.

## Role of beta-blocker therapy

The role of beta-blocker therapy has not been extensively studied in patients with HFpEF. The SENIORS trial reported a reduction in all-cause mortality or cardiovascular-related hospitalizations associated with the use of nebivolol vs. placebo. However, the generalizability of this study to patients with HFpEF is limited as only ~15% of participants had a LVEF >50% (11). Coupled with a high discontinuation rate secondary to drug intolerance in the SENIORS trial, it may be best to reserve beta-blocker therapy for patients with alternative indications where there is proven clinical benefit.

## Role of aldosterone antagonists therapy

The role of spironolactone in HFpEF was assessed in the Aldo-DHF trial, which demonstrated an improvement in diastolic function (reported as an estimate of filling pressure), but not maximal exercise capacity at 12 months compared with placebo and the clinical relevance remained in question (12). The TOPCAT trial found no difference in the composite primary outcome of cardiovascular death, cardiac arrest and HF hospitalizations, but did find a reduction in the incidence of HF-related hospitalizations (13). Notably, this study included patients with EF ≥45%, meaning that the benefit of spironolactone was not exclusive to those with EF ≥50%. While the evidence to support use of an aldosterone antagonist in HFpEF is weak, given most patients in TOPCAT had HFpEF, the plausible reduction in HF hospitalization in this population, and the low medication cost, initiation of spironolactone in patients with HFpEF may be reasonable.

## Role of SGLT2I therapy

The EMPEROR-PRESERVED trial demonstrated fewer events in the composite outcome of cardiovascular death and HF hospitalization with empagliflozin vs. placebo, regardless of diabetes. However, this effect was driven by the reduced incidence of HF-related hospitalizations with zero difference in all-cause mortality (14). This is yet another trial that did not exclusively study patients with EF ≥50%. Although a reduction in hospitalizations would otherwise support the initiation of SGLT2I therapy in patients with HFpEF, several patients included in the EMPEROR- PRESERVED trial had HFmrEF with subgroup analysis illustrating attenuation of this benefit as EF increased. Furthermore, the benefit among those with HFpEF may be offset by the high-cost of SGLT2I agents. In accordance with this discussion, the recently published 2022 AHA/ACC/HFSA guideline suggests that SGTL2-I can be beneficial in patients with HFpEF (2b; moderate strength recommendation and quality of evidence) (5).

## Miscellaneous therapies

Additional trials have evaluated whether medications from other therapeutic classes play a role in the management of HFpEF. Digoxin was evaluated in the DIG-PEF trial and whilst a potential reduction in the composite outcome of mortality and hospitalizations was observed at 2 years, the benefit was not sustained at the conclusion of the study (37 months) (15). Cyclic guanosine monophosphate pathway stimulators were studied in various trials (INDIE-HFpEF, VITALITY- HF-pEF, CAPACITY-HFpEF, and NEAT-HFpEF), but failed to show an increase in either exercise tolerance or quality of life (16–19). Lastly, the phosphodiesterase-5 inhibitor, sildenafil was studied in the RELAX trial, but also failed to show any benefit in exercise tolerance (20).

## Conclusion

The pharmacological management of HFpEF continues to be an area of uncertainty due to multiple studies failing to show a clear benefit associated with therapies that have otherwise proven useful in the management of other HF subtypes. Currently, there are no approved medications that have demonstrated improved survival in patients with HFpEF. The initiation of therapies such as ACEI/ARB or beta-blockers is primarily based on their efficacy in the management of other comorbid conditions. The 2021 ESC guideline update recommends that “in the absence of recommendations regarding disease-modifying therapies, treatment should be aimed at reducing symptoms of congestion with diuretics.” Likewise, the strong recommendation in the recently published 2022 AHA/ACC/HFSA guideline for use of diuretics, as needed in this patient population are concordant with those in the 2021 ESC guideline.

One of the most significant limitations in the current literature is the lack of data pertaining to those exclusively with EF ≥50% and future studies should aim to recruit only patients who meet this definition of HFpEF. In order to best evaluate the clinical implications of these therapies, studies should attempt to measure outcomes such as mortality, hospitalization, exercise tolerance and quality of life with adequate duration of follow up to ensure that any benefits seen early on are sustained. As we continue to search for therapies that may provide mortality/morbidity benefits to those with HFpEF, it is important for us as clinicians to understand the results and limitations of these pivotal clinical trials as they continue to emerge in order to make informed decisions in the interest of balancing risks and benefits to our patients. Until then, I guess we will stick with diuretics as our mainstay of therapy.

## Author contributions

Information was gathered by both LT and LH. Both authors have read and agreed to the content of the manuscript.

## Conflict of interest

The authors declare that the research was conducted in the absence of any commercial or financial relationships that could be construed as a potential conflict of interest.

## Publisher's note

All claims expressed in this article are solely those of the authors and do not necessarily represent those of their affiliated organizations, or those of the publisher, the editors and the reviewers. Any product that may be evaluated in this article, or claim that may be made by its manufacturer, is not guaranteed or endorsed by the publisher.
